# Predictive and Prognostic Relevance of Tumor-Infiltrating Immune Cells: Tailoring Personalized Treatments against Different Cancer Types

**DOI:** 10.3390/cancers16091626

**Published:** 2024-04-23

**Authors:** Tikam Chand Dakal, Nancy George, Caiming Xu, Prashanth Suravajhala, Abhishek Kumar

**Affiliations:** 1 Genome and Computational Biology Lab, Department of Biotechnology, Mohanlal Sukhadia University, Udaipur 313001, Rajasthan, India; 2 Department of Biotechnology, Chandigarh University, Mohali 140413, Punjab, India; nancy.george@cumail.in; 3 Department of Molecular Diagnostics and Experimental Therapeutics, Beckman Research Institute of the City of Hope, Monrovia, CA 91010, USA; caixu@coh.org; 4 Amrita School of Biotechnology, Amrita Vishwa Vidyapeetham, Clappana P.O. 690525, Kerala, India; prash@bioclues.org; 5 Manipal Academy of Higher Education (MAHE), Manipal 576104, Karnataka, India; 6 Institute of Bioinformatics, International Technology Park, Bangalore 560066, Karnataka, India

**Keywords:** cancers, tumor microenvironment (TME), tumor-infiltrating immune cells (TIICs), immunotherapy, predictive biomarkers, prognostic biomarkers, precision medicine, personalized treatment

## Abstract

**Simple Summary:**

This review summarizes the pivotal role of tumor-infiltrating immune cells (TIICs) within the tumor microenvironment (TME) and their impact on cancer prognosis and treatment response. By analyzing TIICs alongside tumor mutation burden (TMB) and immune checkpoint inhibitor (ICI) scores, this study reveals insights into cancer’s immune landscapes. Understanding TIICs’ influence enables tailored cancer treatments, aiding postoperative care, therapy decisions, and personalized medicine choices. We effectively examined the predictive and prognostic value of TIICs alongside TMB and ICI scores in identifying the diverse immunological environments of cancer. Several approaches discussed in this review provide more accurate predictions of patient outcomes and treatment responses. These models can help identify individuals who may derive greater benefit from adjuvant or neoadjuvant treatment. In summary, we believe that the major contribution of TIICs in cancer will have a substantial positive impact on postoperative follow-up, therapy, interventions, and the ability to make educated decisions regarding personalized cancer treatments. This comprehensive study underscores the significant role of TIICs in combating tumor-mediated immunosuppression and fostering antitumor immune responses, promising improved cancer prognosis and therapeutic outcomes.

**Abstract:**

TIICs are critical components of the TME and are used to estimate prognostic and treatment responses in many malignancies. TIICs in the tumor microenvironment are assessed and quantified by categorizing immune cells into three subtypes: CD66b+ tumor-associated neutrophils (TANs), FoxP3+ regulatory T cells (Tregs), and CD163+ tumor-associated macrophages (TAMs). In addition, many cancers have tumor-infiltrating M1 and M2 macrophages, neutrophils (Neu), CD4+ T cells (T-helper), CD8+ T cells (T-cytotoxic), eosinophils, and mast cells. A variety of clinical treatments have linked tumor immune cell infiltration (ICI) to immunotherapy receptivity and prognosis. To improve the therapeutic effectiveness of immune-modulating drugs in a wider cancer patient population, immune cells and their interactions in the TME must be better understood. This study examines the clinicopathological effects of TIICs in overcoming tumor-mediated immunosuppression to boost antitumor immune responses and improve cancer prognosis. We successfully analyzed the predictive and prognostic usefulness of TIICs alongside TMB and ICI scores to identify cancer’s varied immune landscapes. Traditionally, immune cell infiltration was quantified using flow cytometry, immunohistochemistry, gene set enrichment analysis (GSEA), CIBERSORT, ESTIMATE, and other platforms that use integrated immune gene sets from previously published studies. We have also thoroughly examined traditional limitations and newly created unsupervised clustering and deconvolution techniques (SpatialVizScore and ProTICS). These methods predict patient outcomes and treatment responses better. These models may also identify individuals who may benefit more from adjuvant or neoadjuvant treatment. Overall, we think that the significant contribution of TIICs in cancer will greatly benefit postoperative follow-up, therapy, interventions, and informed choices on customized cancer medicines.

## 1. Introduction

Both the adaptive and innate immune responses have a role in tumor immunosurveillance and the development of cancer [1]. Cells that participate in circumventing tumor-mediated immune suppression typically exhibit an inflammatory phenotype and are responsible for reinvigorating the anti-tumor immune response [2,3]. Nevertheless, it is worth noting that immune cells can also facilitate the proliferation and maturation of the neoplasm. This phenomenon can be attributed to the immunosuppressive properties exhibited by various immune cells associated with tumors. The facilitation of angiogenesis, cell proliferation, and tissue remodelling, as well as the encouragement of an anti-inflammatory immune response, result in cancer-related phenotypes [1,3,4]. Within the tumor microenvironment, there exists a dynamic interplay between TIICs, stromal (or intraepithelial) cells, cancer-associated fibroblasts (CAFs), and cancer cells, which serve to regulate the progression of cancer [5,6,7]. The composition of stromal cells exhibits variation across different tumor types, encompassing endothelial cells, fibroblasts, adipocytes, and stellate cells. TIICs are critical in the origin and progression of varied cancer types [6,8,9,10]. TIICs encompass immune cells, such as T-cells, B-cells, natural killer (NK) cells, neutrophils, and macrophages that undergo migration from the bloodstream into the tumor [11,12,13]. M1/M2 macrophages, N1/N2 neutrophils, CD4+ T cells (T-helper), CD8+ T cells (T-cytotoxic), FOXP3+CD4+ Tregs, and antigen-presenting DCs are widely known infiltrating immune cells and are implicated in a spectrum of tumor pathologies [14,15]. Tumor-infiltrating lymphocytes (TILs) have been extensively investigated as a prominent subset of TIICs, and earlier studies have documented their association with positive prognostic outcomes in breast [16,17,18], pancreatic [19], urological [20], ovarian [21], lung [12] and many other cancers [22,23]. TIICs serve a crucial role in aiding both the pro- and anti-tumor immune responses to cancer. Notably, the quantities of these cells are indicative of treatment efficacy, overall survival, and other prognoses. However, the intricate relationship between TIICs and their predictive and prognostic relevance in different cancers is largely unknown.

The first and foremost objective of this review is to study the intratumoral immune profile of infiltrating immune cells and assess their clinical implications to develop a unique immunological categorization for a cancer-specific immunotherapy response. T cell markers such as CD3, CD4, and CD8 are frequently utilized markers, with each marker fulfilling distinct functions within the context of normal physiological settings [7,15]. The presence of CD4+ T lymphocytes infiltrating tumors has been correlated with unfavorable relapse-free survival outcomes in cases with translocation renal cell carcinoma (RCC) [24]. Elevated levels of CD8+ T cells have been found to be correlated with unfavorable outcomes in various types of cancer, including prostate cancer, clear cell renal cell carcinoma (RCC), Hodgkin lymphoma, and follicular lymphoma [25,26,27,28,29]. In a prior investigation on breast cancer, it was observed that individuals exhibiting elevated levels of CD8+ T lymphocytes near the invasive margins of tumors have a worse prognosis after a period of 14 years [30]. Nevertheless, the densities of CD8+ T cells in the tumor did not show any correlation with the prognosis of breast cancer [30]. In summary, CD8+ T lymphocytes have the potential to yield conflicting prognostic forecasts across various tumor locations, as indicated by previous studies [30]. Furthermore, a lack of consensus also exists on the roles of B lymphocytes (CD20+) and NK cells (CD57+) in the context of malignancy [31]. TAMs play a crucial role as immune cells within the tumor microenvironment [32]. TAMs can be classified into two primary subtypes: M1 and M2 [33,34,35]. CD68 serves as a pan-macrophage marker, being expressed in both M1 and M2 macrophages [36]. Conversely, CD163 exhibits unique expression solely in M2 macrophages [37]. An association has been established between heightened CD163+ macrophage density and a less favorable prognosis in patients with cervical, ovarian, breast, and bladder malignancies [38,39,40,41,42]. Additionally, tumor-associated macrophages (TAMs) represent an immune cell subset capable of influencing the interaction between cancer cells and the immune system. Previous clinicopathological studies have suggested that an increase in TAMs often correlates with an unfavorable clinical prognosis [10]. Collectively, these findings indicate that the distribution patterns of various immune cell markers, including CD3, CD4, CD8, CD20, CD57, CD68, and CD163, hold potential as prognostic indicators for individuals diagnosed with cancer.

Furthermore, we assess how these markers may also assist in assessing the efficacy of adjuvant therapy or neoadjuvant therapy. In individuals diagnosed with malignancies that express human epidermal growth factor receptor 2 (HER2), a substantial presence of TILs has been correlated with enhanced overall survival (OS) rates. Moreover, this high TIL count may serve as an indicator of a more favorable response to anthracyclines and trastuzumab, as suggested by previous studies [43,44,45,46]. Furthermore, it has been observed that the presence of TILs can lead to a reduction in distal recurrence and an improvement in metastasis-free survival in patients with previously untreated triple-negative breast cancer, as indicated by studies conducted between the years 2011 and 2014. Nevertheless, it is worth noting that the quantity of TILs does not consistently correlate with the efficacy of treatment, indicating that the immunological characteristics of TILs (qualitative traits) play a significant role in predicting outcomes [17]. Moreover, there exists a lack of consensus about the impact of TIICs on individuals with cancer concerning therapeutic responsiveness, overall survival, disease free survival and others. This discrepancy in viewpoints could potentially be attributed to variations in the molecular subtypes of tumors across stages of tumor progression [17,47,48]. The objective of this review is to explore the extent to which immunological and inflammatory cells that infiltrate tumors accurately reflect the immune microenvironment, and to assess their potential clinicopathological effects towards development of successful cancer therapeutics.

## 2. Technological and Computational Interventions for Assessment and Quantification of TIICs

TIICs are essential constituents of the tumor microenvironment and have been employed for prognostic and therapeutic purposes in individuals with cancer. Quantification of TIICs may reveal the immune system’s complex function in human malignancies, tumor escape mechanisms, and therapeutic response. Several computational algorithms and bioinformatics approaches can aid in the quantification of TIICs. Only a single review paper is available in the literature that comprehensively describes different platforms available to quantify immune cells and cancer cells [49].

### 2.1. Conventional Approaches and Their Limitations

Stratifying cancers via intratumoral immune cell infiltration is promising [50,51,52,53]. The composition of human cancer immune infiltrates has been studied using immunohistochemistry (IHC), immunological fluorescence (IF), and flow cytometry [49]. The IHC immuno-scoring method has two drawbacks. First, pathologists and institutions interpret immune cell subsets differently, making scores inconsistent. Second, only a few indicators can be examined simultaneously, preventing a complete tumor microenvironment (TME) immune contexture annotation. Many reasons may explain these contradictory results related to the influence of TIIC infiltration on cancer and its prognosis. Only one or two types of TIICs with a small sample size [54,55,56,57] or a study using a single bioinformatics method without experimental validation may also lead to incorrect results [58,59]. Thus, effective genome data-informed cell type quantification approaches are urgently needed. Researchers have explored the state-of-the-art computational methods for quantifying immune cells from transcriptomics data and explained potential difficulties that must be solved to reliably quantify immune infiltrates from human bulk tumor RNA sequencing data and others [49]. Aran and co-authors employed xCell, a unique gene signature-based technique, to infer 64 immune and stromal cell types [60]. They harmonized 1822 pure human cell-type transcriptomes from diverse sources, used curve fitting for linear comparison, and presented a novel spillover compensation technique for separating them. Their detailed in-silico evaluations and comparison to cytometry immunophenotyping reveal that xCell surpasses other approaches. xCell is available at http://xCell.ucsf.edu/, accessed on 2 November 2023.

After the steep advancements in next-generation sequencing (NGS) technologies at affordable costs, it has become considerably encouraging to use NGS in routine oncology as well as in large-scale collaborative efforts—for instance, the Gene Expression Omnibus (GEO) database [61], The Cancer Genome Atlas (TCGA) [62,63], just to name a few. We now have access to an unprecedented amount of RNA sequencing (RNA-seq) data describing the tumor microenvironment. These gene expression profiles and immune cell landscapes can predict clinical outcomes and immunotherapy response. Computational methods based on immune-specific marker genes or expression profiles can characterize TIICs from bulk tumor RNA-seq data. We believe that recently developed state-of-the-art technologies can be applied to publicly available microarray expression data sets and new microarray or RNA-seq-based transcriptome profiles to help understand the microenvironment’s facilitation of neoplastic cell growth and genomic alterations.

### 2.2. Machine Learning-Based Computational Approaches for the Establishment of ICI Scores

In recent decades, significant progress has been made in the field of NGS technology, particularly in the development of NGS algorithms. These advancements have led to the discovery of extensive biological insights into the processes of tumorigenesis and metastasis [64]. Studies employ different computational techniques, namely GSEA (https://www.gsea-msigdb.org/gsea/index.jsp, accessed on 2 November 2023) [65], CIBERSORT (http://cibersort.stanford.edu/, accessed on 2 November 2023) [66], ESTIMATE (https://bioinformatics.mdanderson.org/estimate/index.html, accessed on 2 November 2023) [67], and others, to examine the gene-expression profiles of bulk tumor samples. The most used marker gene analysis method is gene set enrichment analysis (GSEA) [65]. An enrichment score (ES) is high when the genes specific to a cell type are among the top highly expressed in the sample of interest (i.e., the cell type is enriched) and low otherwise. Single-sample GSEA (ssGSEA) calculates an ES indicating the degree to which genes in a gene set are coordinately up-or down-regulated in a single sample [68]. Compared to the original GSEA framework, ssGSEA ranks genes by their absolute expression in a sample and computes ES by integrating the empirical cumulative distribution functions of the gene ranks to obtain a full understanding of the intratumoral immune landscape [66,67,69].

**Figure 1:**
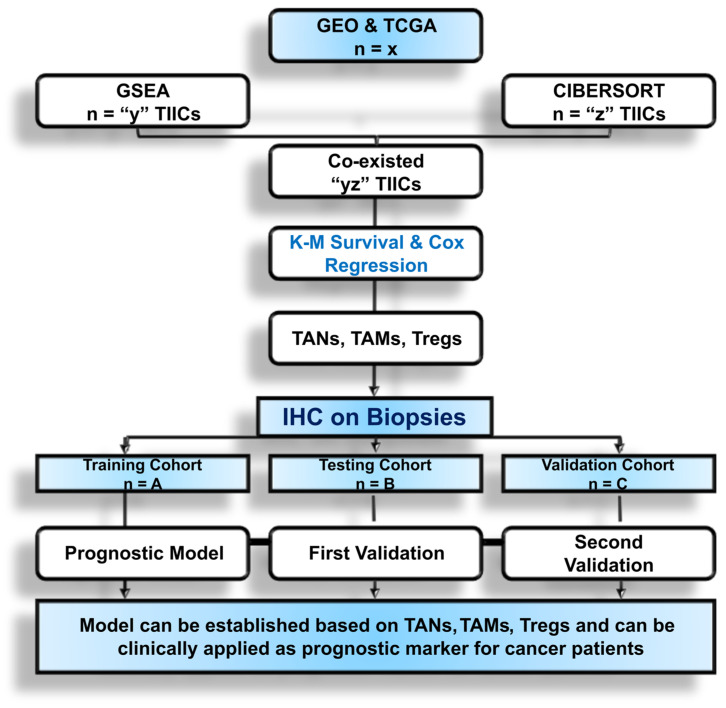
Overview of data tapping for the establishment of the prognostic model for ascertaining TIIC association with cancer using two machine-learning tools followed by IHC on patients’ biopsies. The numbers mentioned are only for reference purposes.

The predictive and prognostic relevance of TIICs can be evaluated using datasets from the GEO [61] and TCGA [63] databases in cohorts specific to any particular cancer type. Three distinct populations of TIICs are generally chosen for IHC validation analysis in a sample of cancer biopsies. These populations include CD66b+ tumor-associated neutrophils (TANs), FoxP3+ Tregs, and CD163+ tumor-associated macrophages (AMs). The link between TIIC composition and cancer prognosis exhibits relationships across several datasets, as determined by the use of single-sample gene set enrichment analysis (ssGSEA) and CIBERSORT analysis [66,67,69]. The central aim of IHC findings is to demonstrate a substantial correlation between TANs, Tregs, TAMs, and the prognosis of cancer patients [69,70]. Predictive and prognostic models can be developed using the training cohort, which consists of certain numbers of patients, to investigate the impact of these TIIC populations on the clinical characteristics and prognosis of cancer patients (Figure 1). The models underwent additional testing and validation in cohorts consisting of certain numbers of cancer individuals (Figure 1). The prognostic value of the infiltrating immune cells can be assessed using univariate analysis.

Least Absolute Shrinkage and Selection Operator (LASSO) regression can be employed for the identification of immune cells that have the most relevance to survival outcomes. The construction of an immune-cell characteristic score (ICCS) model will then be achieved by the application of multivariate Cox regression analysis. Furthermore, these correlations are also observed in disease-free survival (DFS) outcomes and OS statistics within the same dataset. These models demonstrate a higher level of reliability (C-index DFS & OS, AIC DFS, AIC OS) as compared to conventional indicators in assessing the prognosis of cancer [69]. Furthermore, these parameters are also evaluated for the independent prognostic indicators based on the statistical analysis—for instance, *p*-values for disease-free survival and overall survival. Finally, the prognostic predictive models were developed using the quantities of TANs, Tregs, and TAMs. The utilization of ssGSEA or CIBERSORT techniques can successfully facilitate prognostic evaluation through the identification of a substantial presence of TIICs [66,67,69]. In summary, there exists a correlation between TIICs and the clinical characteristics and prognosis of individuals diagnosed with cancer, hence suggesting the potential utility of TIICs as biomarkers.

Deconvolution methods can quantify the relative fractions of cell types of interest, unlike GSEA-based approaches that can only compute a semi-quantitative score describing cell type enrichment in a sample [49]. Hao et al. identified the problem that outliers in gene-expression data often lower estimation accuracy [71]. Therefore, a reliable deconvolution approach that detects and removes outliers is needed to cleanse data automatically. A signature matrix describing cell-type-specific expression profiles can be used by deconvolution algorithms to estimate unknown cell fractions from heterogeneous sample gene expression profiles [49]. Multiple dataset heterogeneity improves cell-mixture deconvolution accuracy and eliminates biological and technical biases [72].

### 2.3. Spatially Variant Immune Infiltration Scoring using SpatialVizScore

The Immunoscore quantifies cancer immune cell infiltration to predict prognosis [73]. Previous immune profiling methods used a few immunological markers to determine tumor immunity. Immune cells are more complex than immunohistochemistry can detect. Allam and co-authors introduced SpatialVizScore, a spatially variable immune infiltration score, to measure immune cell infiltration in lung tumor samples using multiplex protein imaging data [73,74]. Imaging mass cytometry (IMC) targeted 26 tumor markers to determine stromal, immunological, and cancer cell states in 26 lung cancer tissues [74]. Unsupervised clustering algorithms used high-dimensional analysis of 16 immune markers and other cancer and stroma-enriched labels to profile tumor immune infiltration patterns. Spatially resolved tumor maps showed immune-cancer cell pairs’ closeness and neighborhoods. SpatialVizScore maps patients’ tumors by immune inflamed, immune repressed, and immune cold states, showing the tumor’s immune continuum allocated to three infiltration score ranges. Cell-based scoring methods at the single-cell and pixel levels depicted the cellular spectra in distinct lung tissues using several inflammatory and suppressive immune markers. Thus, SpatialVizScore is a new quantitative tool for studying cancer tissue tumor immunology.

### 2.4. Nonnegative Tensor Factorization using ProTICS

Distinct genetic profiles and targeted therapies are often essential for various subtypes within a single cancer type. Variations in the cellular and molecular features of the tumor microenvironment among different cancer subtypes significantly impact tumor progression and prognostic outcomes. Despite extensive research on the prognostic relationship between TILs and specific histological subtypes, there is a dearth of comprehensive studies systematically investigating the prognostic role of immune cells in molecular subtypes. This gap includes the underutilization of machine learning methodologies for analyzing multi-omics datasets. Liu et al. (2021) introduced an innovative computational framework, ProTICS, designed to quantitatively evaluate shifts in immune cell distributions within the TME and forecast their prognostic significance across diverse subtypes [75]. Initially, patients were categorized into distinct molecular subgroups by the utilization of gene expression and methylation profiles. This was achieved by employing the nonnegative tensor factorization technique [75]. Subsequently, the proportion of cell types in each specimen was measured utilizing an mRNA-based deconvolution methodology. Cox proportional hazard regression was utilized to determine the prognostic impact of immune cell types for cancers in each subtype. At the molecular level, they made prognostic predictions for hallmark genes associated with each subtype. Ultimately, they conducted a performance evaluation of ProTICS on three TCGA datasets and one additional independent METABRIC dataset. This approach successfully classified cancers into numerous molecular subtypes, each distinguished by significant variations in overall survival rates. Additionally, the study revealed distinct prognostic patterns of different immune cell types across molecular subtypes. This investigation provided fresh insights into the prognostic relationship between immune cells and molecular subtypes, highlighting the potential of immune cells as prognostic markers [75]. The ProTICS is publically available and the R code can be accessed at the GitHub repository: https://github.com/liu-shuhui/ProTICS (accessed on 2 November 2023) [75].

## 3. Heterogeneity in the Predictive and Prognostic Relevance of TIICs in Different Cancer Types

The TME comprises diverse cellular populations, encompassing malignant, nonmalignant, immune, and stem cells, which facilitate tumor growth, invasion, and metastasis through intricate communication networks [5,7,11,76]. The composition of the immune microenvironment within a tumor plays a crucial role in determining both patient survival outcomes and their response to immunotherapy interventions [77,78]. Zou and co-authors aimed to identify the patterns of immune cell infiltration in 32 different forms of cancer [79]. It was shown that patients belonging to the high immune cell infiltration cluster exhibited poorer OS rates, but experienced better PFI in comparison to those in the low immune cell infiltration cluster. Nevertheless, the predictive value of immune cell infiltration varied among different types of cancer [79]. Elevated levels of immune cell infiltration (referred to as High CI) are associated with a poorer prognosis in brain lower-grade glioma (LGG), glioblastoma multiforme (GBM), and uveal melanoma (UVM) [79]. Conversely, a higher immune cell infiltration has been linked to a more favorable prognosis in adrenocortical carcinoma (ACC), cervical squamous cell carcinoma and endocervical adenocarcinoma (CESC), cholangiocarcinoma (CHOL), head and neck squamous cell carcinoma (HNSC), liver hepatocellular carcinoma (LIHC), lung adenocarcinoma (LUAD), sarcoma (SARC), and skin cutaneous melanoma (SKCM) [79]. The prognosis of lung adenocarcinoma (LUAD) was found to be notably impacted by the presence of 13 distinct immune cell types. A good prognosis was observed when there was a high level of infiltration by all immune cell types, except for Type 2 T helper (Th2) cells. A model known as the ICCS model was developed, which utilizes six immune cell populations that are most critical for survival. This model is capable of categorizing patients into two groups: low-ICCS and high-ICCS. Patients in the low-ICCS group have a favorable prognosis, while those in the high-ICCS group have a negative prognosis. The results obtained from several multivariate and stratified analyses provided additional evidence that the ICCS exhibited independent prognostic significance for different cancer types [79,80,81].

To gain a deeper comprehension of the association between the infiltration of immune cells and the prognosis of tumors, it is critical to assess the survival correlation of the infiltrating immune cells in a cancer or different cancers. In general, there exists a clear association between the presence of invading immune cells and the prognosis of individuals with cancer across many measures such as OS, DSS, progression-free survival (PFS), disease-free survival (DFS, also known as relapse-free survival, RFS), time to treatment failure (TTF), and quality of life (QoL). However, the number of immune cells associated with DFS is relatively low in comparison to OS, DSS, and PFS. In several cancer types, the presence of immune cell infiltration is linked to the prognosis of the disease. Notably, a strong correlation between high B cell infiltration and favorable prognosis has been observed in the majority of cancer types [82,83]. One potential rationale for the observed disparity could be attributed to the omission of the B-cell-rich subtype in the subsequent investigations, which demonstrated a notably immunosuppressive microenvironment [84]. The immunosuppressive substances may potentially restrict the anti-tumor action. Comparably, the presence of PD1+ depleted CD8+ T lymphocytes within the TME has been linked to unfavorable prognostic outcomes in various types of human malignancies [85,86,87,88].

Heterogeneous tumor immune cells show functional and phenotypic flexibility and may promote or inhibit tumor growth. Interestingly, the distribution of immune cell subsets and their exact placement with cancer cells may predict tumor behaviour. The following sections describe the role of different populations of TIICs and TMB and ICI scores in describing the evident heterogeneity in different cancer types.

### 3.1. Predictive and Prognostic Powers of Tumor Infiltrating Lymphocytes: T-Regs, CD4+ T-Helper, CD8+ Cytotoxic T-Cells, and B-Cells

The presence of TILs has the potential to impact the progression of cancer. Tumor-infiltrating lymphocytes can be broadly categorized as either tumor-suppressive or tumor-promoting lymphocytes. Notably, the abundance of these cells might serve as a predictive indicator of therapy efficacy and overall survival. CD3, CD4, and CD8 are often observed markers on T cells, with each marker fulfilling distinct functions in the absence of any aberrations. For example, CD3+ T cells serve as indicators for the presence of all T cells. CD4+ T cells are commonly referred to as T helper (Th) cells, while mature CD8+ T cells are known as cytotoxic T lymphocytes (CTLs). It is important to mention that CD3+ T cells encompass the entirety of T lymphocytes, while CD4+ T and CD8+ T cells indicate the proportional distribution of their respective subgroups, namely Th cells and CTLs.

The presence of CD4+ T lymphocytes infiltrating tumors has been linked to a reduced likelihood of relapse-free survival in translocation renal cell carcinoma (RCC) [24]. An unfavorable prognosis has been observed in cases of prostate cancer [25], clear cell renal cell carcinoma (RCC) [26,27], Hodgkin lymphoma [28], and follicular lymphoma [29], when there is a high concentration of CD8+ T cells. In a prior investigation on breast cancer, it was observed that individuals who exhibited elevated levels of CD8+ T lymphocytes near the invasive margins (IMs) of tumors experienced an unfavorable prognosis after 14 years. Nevertheless, the densities of CD8+ T cells in the tumor center (TC) did not exhibit any significant correlation with the prognosis of breast cancer. In brief, it has been observed that CD8+ T lymphocytes can yield conflicting prognostic forecasts across various tumor areas [30].

**Figure 2:**
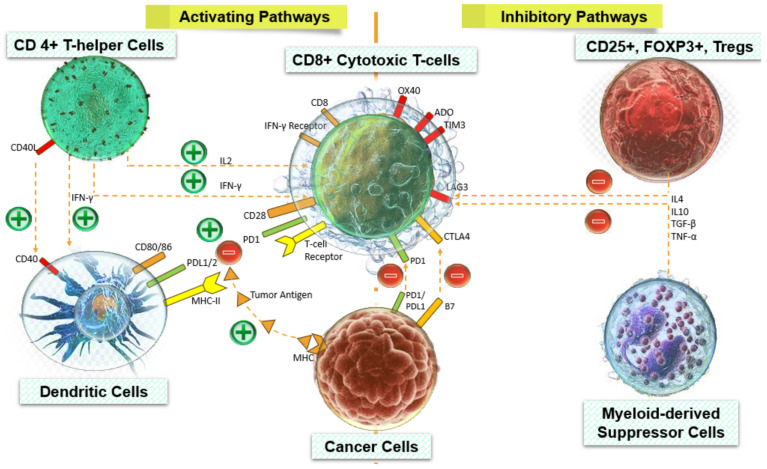
Pathways are activated and inhibited by infiltrated immune cells in tumors.

Cytotoxic CD8+ T cells are crucial in maintaining anti-cancer immunity through their direct targeting of cancer cells via FAS-mediated apoptosis and perforin-mediated cytolysis [89]. Tregs have been identified as the primary lymphocytes responsible for tumor growth within the TME [90]. Regulatory T cells exert inhibitory effects on the anti-cancer functions of CD8+ T cells, as well as CD4+ T cells and dendritic cells (DCs) that facilitate the activation of CD8+ T cells (Figure 2). Tregs utilize a variety of contact-dependent and cytokine-mediated pathways to achieve this objective, as extensively examined by Han and co-authors [91].

As an illustration, the expression of perforin and granzyme by Tregs within the TME, as opposed to naïve Tregs (Figure 2), initiates the process of lysing effector T cells and NK cells [92]. Furthermore, the expression of CD39 and CD73 on Treg cells facilitates the enzymatic conversion of adenosine, hence leading to the suppression of the anti-tumor activity exhibited by other T cells [93]. Hence, it is unsurprising that CD8+ and regulatory T cells are widely employed as indicators for cancer prognosis. This review offers a potential rationale for the contrasting associations observed between intratumoral Tregs and overall survival in various cancer types. Additionally, the current review presents straightforward guidelines for the identification of optimal prognostic biomarkers, considering the diverse occurrence and role of intratumoral lymphocytes.

This section deals with the dynamic conversion processes and immunomodulatory effects of B cells in the context of different cancers. The regulation of B cell invasion, development, and polarization is influenced by the tumor microenvironment [12]. B cells can impede the progression of tumors through various mechanisms. These include the secretion of immunoglobulins, which can enhance the response of T cells and potentially induce direct tumor cell death. In addition, B lymphocytes that are activated by lung tumors release immunoglobulins that can facilitate tumor destruction through mechanisms such as antibody-dependent cellular cytotoxicity (ADCC) or complement-dependent cytotoxicity (CDC). However, it is important to note that B cells can also exert a suppressive effect on the antitumor immune response. This is primarily mediated by a subset of B cells known as regulatory B cells (Bregs). Bregs can produce immunosuppressive cytokines that regulate the activity of T cells, NK cells, and myeloid-derived suppressor cells (MDSCs). Hence, the correlation between the density of tumor-infiltrating B-lymphocytes (TIBs) and the activation of CD8+ T and CD56+ NK cells inside the tumor microenvironment is evident. This relationship potentially contributes to the augmentation of the local antitumor immune response and indicates a favorable prognosis [94]. Additionally, Bregs can secrete antibodies that contribute to pathological processes and facilitate the formation of new blood vessels (angiogenesis). Furthermore, the density of TIBs exhibited a correlation with the heightened production of granzyme B and IFN-1, both of which serve as indicators of activation in cytotoxic T and NK cells.

#### 3.1.1. CD68 is a Pan-Macrophage Marker Expressed in M1 and M2 Macrophages, while CD163 is M2-Specific

Different subtypes of myeloid cells have an impact on the immunological microenvironment. While there have been findings that showed favorable prognosis in certain studies [95,96], several investigations have consistently demonstrated a significant association between a high abundance of tumor-infiltrating macrophages and unfavorable prognosis in different types of cancers [55,97,98]. Macrophages exhibit heterogeneity and can be categorized into two main subtypes: classically activated macrophages (also known as M1 macrophages or M1 as discussed earlier) that are primarily activated by Th1 cytokines, and alternatively activated macrophages (also known as M2 macrophages or M2) that are primarily activated by Th2 cytokines [99,100,101,102]. It is postulated that the observed inconsistency in prior findings could perhaps be attributed to the methodology employed in earlier research, wherein the assessment of tumor-infiltrating macrophages was conducted by considering them as a whole or as M2 macrophages, rather than independently evaluating M1 macrophages. M1 macrophages exhibit potent tumoricidal action, which stands in contrast to the effects exerted by M2 macrophages. M2 macrophages predominate within the TME, whereas M1 macrophages are primarily found in the non-cancerous inflammatory zone surrounding the cancer cell infiltrates. Hence, it is crucial to evaluate the infiltration of macrophages by individually analyzing the tumor-infiltrating M1 and M2 phenotypes, as well as their respective spatial distributions.

CD204-expressing M2 macrophages have been identified as prognostic indicators (both favorable or unfavorable prognosis) in various types of cancer, including urothelial cell carcinoma of the BC [103], ESCC [104], PDAC [105], and NSCLC [106]. A comparable pattern of prognostic significance was seen, underscoring the significance of CD204+ M2 macrophages inside the TME in thymic carcinoma [105]. A study, which included a sample size of approximately 200 cases of pancreatic ductal carcinoma (PDC), revealed significant positive connections between tumor-infiltrating pan-macrophages, specifically CD163+ or CD204+ M2, and Neu [107,108,109].

#### 3.1.2. Predictive and Prognostic Relevance of Other Immune Cells: Neutrophils, Eosinophils, Mast Cells, and CAFs

**Figure 3:**
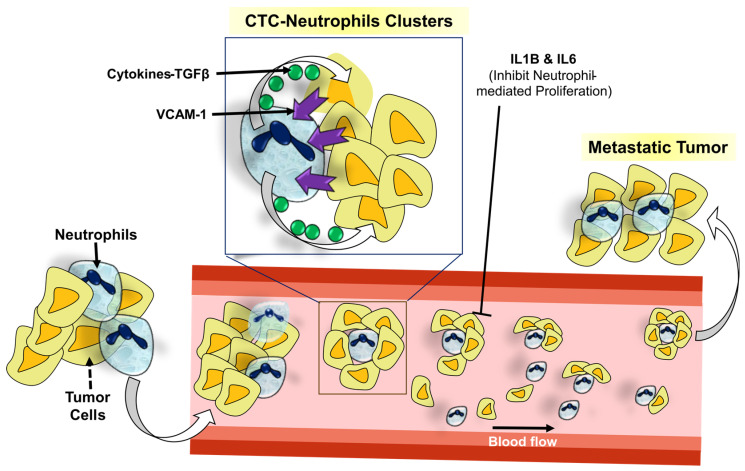
Tumor infiltration by neutrophils. Neutrophils form clusters with circulating tumor cells, circulate through blood streams, and contribute to tumor metastasis at distant locations.

The prognostic relevance of infiltrating innate immunity-related cells, including NK cells, MDSCs, macrophages, and DCs, differed significantly among different tumor types. Various types of immune and inflammatory cells that infiltrate tumors, including Tregs, MDSCs, and alternatively activated macrophages, play a role in promoting tumor growth and progression. These cells achieve this by suppressing the immune responses of the host (Figure 3), facilitating the formation of new blood vessels (angiogenesis), and promoting changes in the structure and composition of the surrounding tissue (tissue remodeling) [4,99,110,111,112]. The prognostic performance of infiltrating Tregs showed heterogeneity across a diverse range of cancers (Figure 3). The outcomes of various malignancies are associated with eosinophils [113], mast cells [114], and neutrophils [115,116]. Specifically, a high presence of mast cells [117] and neutrophils [115,116] has been linked to a negative prognosis, whereas the infiltration of eosinophils has been indicative of a favorable prognosis [113].

**Figure 4:**
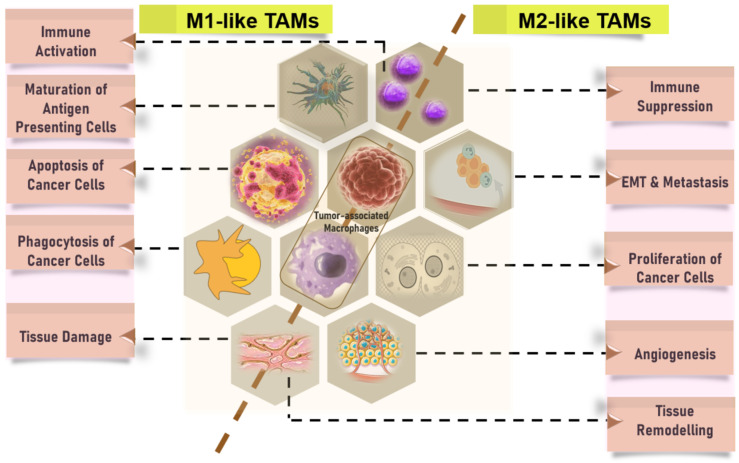
Predictive and prognostic relevance of M1-like (left panel) and M2-like TAMs (right panel) in cancers. M2-like macrophages secrete adrenomedullin and VEGFs to promote angiogenesis. They also release immunosuppressive molecules such as IL10, PD-L1, and TGFβ, which promote tumor growth. Cancer cells see them as “friendly entities“. Tregs, MDSCs, and, alternatively, activated macrophages suppress host immune responses and accelerate angiogenesis, EMT, and tissue remodelling, supporting tumor growth and metastasis.

Cancer cells can attract neutrophils, also known as TANs, which then release NETs into the tumor microenvironment (Figure 4). NETs have been detected in diverse specimens of both human and animal neoplasms, including but not limited to pancreatic, breast, liver, and gastric malignancies, as well as in the vicinity of metastatic tumors [118]. The involvement of neutrophil extracellular traps (NETs) in the progression of tumors is becoming more recognized, particularly with cancer immunoediting and the interplay between the immune system and cancer cells [118]. Based on the available body of information, it has been shown that NETs can activate quiescent cancer cells, leading to the recurrence of tumors, as well as uncontrolled proliferation and metastasis (Figure 4). NETs have a significant regulatory function within the tumor microenvironment, particularly concerning the formation of distant metastases. This is achieved by the release of proteases, namely matrix metalloproteinases, as well as proinflammatory cytokines [118].

In the majority of cancers, a notable correlation was observed between a heightened concentration of CAFs inside the tumor microenvironment and unfavorable clinical outcomes [115]. Collectively, the invasion of immune cell populations demonstrated varied prognostic outcomes across different forms of cancer.

#### 3.1.3. The Prognostic Value of TMB and the Relationship between TMB and Tumor Immune Infiltration

The TMB can predict immune checkpoint inhibitor response in several cancer types. However, it may predict some cancers better than others. Laboratory tests using next-generation sequencing of tumor samples can estimate the TMB by looking for a wide spectrum of mutations. Though less established than measuring TMB from tumor tissue biopsy samples, research is now examining TMB from plasma tumor DNA, which could lead to blood testing in the future. The number of mutations per megabase (mut/Mb) in a DNA region is the TMB. In cancer treatment, tumor mutational burden (TMB) is a new biomarker. The index shows the number of mutations per megabase (muts/Mb) in tumor cells in a certain neoplasm [119]. TMB is high if it reaches 17–20 muts/Mb [120]. Recent investigations have shown that this cut-off may vary substantially depending on tumor type. Marabelle and co-authors (2020) employed 10 muts/Mb for solid tumors [121], while Schrock and co-authors (2019) found 37 muts/MB for colorectal malignancies [122]. Samstein and co-authors (2019) recommended that the ideal TMB-high group overlap with each histology’s highest mutational burden quintile [123]. TMB is a prognostic biomarker for immunotherapy response at high values [124]. TMB-high cancers may respond better to immune checkpoint inhibitors, which engage the immune system to detect cancer cells. Clinical trials are underway to determine which high-TMB tumors respond best to immune-boosting medicines. The immune checkpoint inhibitor pembrolizumab is approved for treating adults and children with advanced malignancies with a high TMB (≥10 mut/Mb) after other treatments have been tried.

Three immune cell infiltration (ICI) patterns are generally established, and the ICI scores are computed by the utilization of principal-component analysis [125]. A high ICI score was associated with an elevated tumor mutation burden (TMB) and heightened activation of immune-activating signalling pathways [125,126]. The low ICI score subtypes exhibited activation of the transforming growth factor-β (TGF-β) and WNT signalling pathways, suggesting the inhibition of T cell activity [126]. This observation may potentially contribute to the unfavorable prognosis associated with these subtypes. Cohorts of patients undergoing immunotherapy have confirmed that those with higher ICI scores exhibit notable therapeutic benefits and clinical advantages [127]. The findings of this study provide evidence supporting the utility of ICI scores as a reliable prognostic biomarker and predictive indicator for immunotherapy. To examine the immunotherapeutic benefits of the ICI score, the immunophenotyping score (IPS) is employed. The expansion of our comprehension of the TME and its implications for immunotherapeutic approaches in any cancer can be achieved by analyzing the ICI patterns in a broader range of samples. This endeavor has the potential to guide ongoing research investigations in the field.

The biological reason for this notion is that tumor cells with a high-TMB produce more immunogenic neoantigens, which host T cells, especially T cytotoxic lymphocytes, recognize to anticipate immunotherapy response. A pooled investigation of 27 tumor types found that anti-PD-1 treatments are the only immunotherapies that respond to high-TMB [128,129]. Intriguingly, tumor cell PD-L1 expression and microsatellite instability (MSI) are both predictive biomarkers of immunotherapy response. As reviewed, a high-TMB can exist without these other biomarkers, suggesting that TMB determination may increase the population who may benefit from immunotherapy [128,129,130,131,132]. However, additional research is needed to answer some unanswered problems, such as: (i) When should immunotherapy be given to cancer patients with high-TMB? (ii) Does TMB assessment help identify immunotherapy responders in MSI/dMMR cancer? (iii) Which therapy option is better for high-TMB with other actionable alterations?

## 4. Common Prognostic Biomarkers for the Presence of Immune Cell Infiltration across Different Cancers

Despite widespread intratumor heterogeneity, some common biomarkers for the presence of tumor immune cell infiltration have been identified in several pan-cancer analyses [133,134,135,136]. In some studies, the expression level of Proteasome Subunit beta 8 (PSMB8) [133], Lipocalin 2 (LCN2) [134], T Cell Immunoreceptor with Ig and ITIM Domains (TIGIT) [135], High Mobility Group Box (HMGBs) proteins [136], and others were found to be greater in tumor tissue as compared to normal tissue [134]. An increased expression of LCN2 was found to be associated with unfavorable clinical outcomes in terms of overall survival (OS) and recurrence-free survival (RFS) [134]. Strong positive associations were seen between the expression of LCN2 and many types of TIICs, such as CD8+ T cells, CD4+ T cells, B cells, neutrophils, macrophages, and dendritic cells [134]. Additionally, markers associated with TIICs displayed distinct patterns of immune infiltration related to Lipocalin-2 (LCN2). The results of the Gene Set Enrichment Analysis (GSEA) indicated a significant association between the expression of LCN2 and retinol metabolism, drug metabolism cytochrome P450, and metabolism of xenobiotics by cytochrome P450 [134]. LCN2 has the potential to function as a biomarker for the presence of immune infiltration and unfavorable prognosis in several types of malignancies, hence providing novel insights into the development of cancer therapies. microsatellite instability (MSI), mismatch repair (MMR) genes, and DNA methyltransferases (DNMTs) were also explored.

Several studies presented novel findings by establishing a noteworthy association between elevated levels of immune gene biomarkers and an unfavorable outcome in patients diagnosed with different cancers. In most cases, expression of a particular immune gene biomarker shows poor prognosis [136,137,138,139,140]; however, in certain cases [141,142], high expression relates to favorable prognostic outcomes (Table 1). Some immune biomarkers showed unfavorable prognosis in certain cancers but showed favorable prognoses (Table 1) in different cancers [136,137,143]. Overexpression of high mobility group box 1 (HMGB1) resulted in poor prognosis in human bladder urothelial carcinoma [143] and patients after radical prostatectomy in prostate cancer [136,137], while its overexpression in gastric adenocarcinomas yields favorable prognosis [144].

## 5. Predictive and Prognostic Relevance Correlate to Activated Signaling Circuits that Rely on Infiltrating Immune Cells

**Figure 5:**
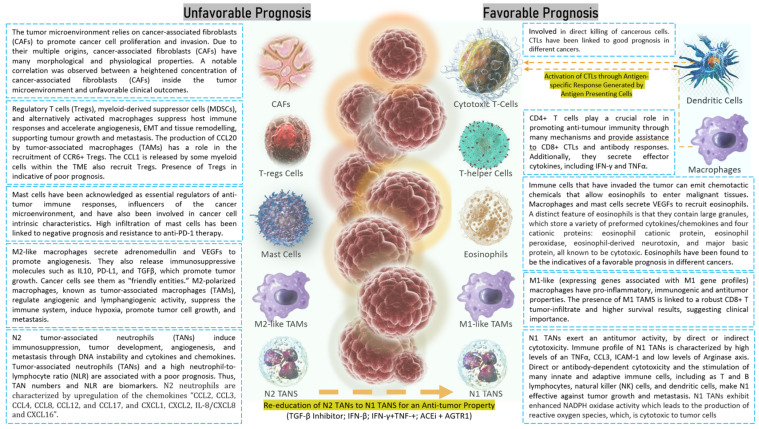
Most important tumor infiltrating immune cells having predictive and prognostic applications. The left panel shows immune cells (such as Tregs, mast cells, M2 TAMS, N2 TANS, CAFs) and the right panel displays immune cells (such as CTLs, Th cells, eosinophils, M1 TAMs, N1 TANs, and antigen presenting cells, dendritic cells, and macrophages).

Expressed immune biomarker are implicated in one or more the cancer-related pathways, for instance the PI3K/AKT signaling system [170,171], TGF-β signaling [170,172], the WNT/β-catenin signaling route [172,173], the NFκB pathway [174], JAK-STAT signalling, RAS signalling, and apoptotic pathways, just to name a few (Figure 5). Infiltrating immune cells along with their biochemical secretions, mainly chemokines, cytokines, and other immunomodulators, have a strong influence on the infiltration of other cells [175]. Often, these molecules exert pro-tumor or anti-tumor effects on immune cells of the TME, leading to the generation of a signaling cascade as a manifestation of the host immune response, which could be inflammatory or anti-inflammatory and immunosuppressive or immunomodulatory (Figure 5). The expression of these pathways has been found to be associated with the infiltration of activated Tregs [15], TANs, TAMs [172,173], myeloid dendritic cells [174], macrophages [36], naive CD4+ T cells, and naive CD8+ T cells and other infiltrating immune cells [170]. IFN-γ is a central player that orchestrates the anti-tumor immune response through activation of the JAK-STAT pathway. On the contrary, a study revealed a positive correlation between elevated ICI scores and the likelihood of immune evasion. This phenomenon has been attributed to the upregulation of the JAK-STAT signaling pathway, correlated to a decrease in the population of CD8+ T cells. This shows surprisingly a non-redundant feature of the JAK-STAT pathway. Likewise, without any contradictions, there is a notable correlation between heightened quantities of TILs, specifically CD4+ T cells and CD8+ T cells, and enhanced rates of survival as well as a heightened response to immunotherapy [172]. A relationship between TOP2A (Topoisomerase IIA) and the emergence and progression of cervical cancer is widely acknowledged. In one study, Wang and co-authors observed an aberrant upregulation of TOP2A in cervical cancer tissues through experimental investigations (transwell invasion and migration, and Western blotting).

Furthermore, they demonstrated that the activation of the PI3K/Akt signaling pathway by TOP2A contributes to cell motility, invasion, and epithelial–mesenchymal transition [171]. As a part of TGF-β signaling, TAMs have been observed to exert several pro-tumor effects through the secretion of immunosuppressive cytokines, including interleukin-10 (IL-10) and transforming growth factor-β (TGF-β) [172]. Additionally, an increase in TAM density is correlated with a worse prognosis [172]. Infiltration of one component of the TME into the tumor can also influence and manipulate the recruitment of others in the tumor. For instance, it has been widely seen that the increased infiltration of stromal components within tumor tissue has the potential to diminish the trafficking of TILs into malignancies. These observations highlight the significance of intercellular interactions within the TME as being more crucial than the individual cellular constituents [176,177]. Altogether, all findings offered a clearer understanding of many possible correlations among TIICs that may contribute to the observed phenotypes. As a result, these insights may have implications for the clinical diagnosis and treatment of cancers, potentially providing new perspectives in this field.

## 6. Relationship between Immune Cells and Cancer Treatment Modalities

The relationship between immune cells and cancer treatment modalities is a fundamental aspect of cancer immunotherapy, an approach that aims to leverage the body’s immune system to recognize and eliminate cancer cells [2,3]. Herein, we have summarized key components illustrating this relationship. Within the intricate network of the immune system, specialized cells constantly survey the body for abnormalities, including cancerous cells. T cells, B cells, NK cells, and DCs are among the key players in this surveillance process. T cells, for instance, can recognize abnormal proteins, or antigens, presented on the surface of cancer cells through a complex mechanism involving antigen-presenting cells like dendritic cells. Upon recognition, T cells become activated and initiate immune responses to eliminate the cancerous threat. Similarly, NK cells are equipped to recognize and directly kill cancer cells without prior sensitization. This ongoing surveillance forms a crucial frontline defense against cancer development and progression.

Cancer cells, however, possess sophisticated mechanisms to evade immune detection and destruction. They can downregulate the expression of antigens, making themselves less recognizable to immune cells, or secrete immunosuppressive factors that inhibit immune cell function. Additionally, cancer cells often exploit checkpoint pathways, such as the PD-1/PD-L1 axis, to suppress immune responses [120]. By engaging with checkpoint molecules, cancer cells can effectively disarm the immune system and evade elimination. This ability to evade immune surveillance is a hallmark of cancer and contributes significantly to tumor growth and spread.

In recent years, immunotherapy has emerged as a promising approach to overcome immune evasion and enhance anti-tumor immune responses. Immune checkpoint inhibitors, for example, work by blocking inhibitory signals that dampen T-cell activity, thereby restoring and enhancing the immune response against cancer cells. Adoptive cell therapy involves the engineering or expansion of immune cells, such as CAR T cells or tumor-infiltrating lymphocytes, to specifically target and kill cancer cells. Cancer vaccines stimulate the immune system to recognize tumor-specific antigens, priming it for an effective anti-tumor response. Additionally, cytokine therapy aims to bolster immune cell function by administering cytokines, such as interleukins or interferons, to the patient.

The TME is a dynamic ecosystem consisting of various cell types, including immune cells, fibroblasts, endothelial cells, and extracellular matrix (ECM) components. Immune cells within the TME play dual roles: they can either promote tumor growth and metastasis or mount anti-tumor immune responses. For instance, TAMs and Tregs often exert immunosuppressive effects, while cytotoxic T cells and NK cells are responsible for directly targeting and killing cancer cells [10,32,69,70]. Moreover, the TME is rich in cytokines and chemokines that regulate immune cell recruitment, activation, and function. The composition and characteristics of the TME greatly influence tumor behavior and response to therapy, highlighting the importance of understanding its complex interplay with immune cells.

Predictive biomarkers are valuable tools for predicting response to immunotherapy and guiding treatment decisions. PD-L1 expression on tumor cells, for instance, has been associated with improved response to immune checkpoint inhibitors in certain cancers. TMB, which reflects the number of mutations present in a tumor’s DNA, has also been identified as a predictive biomarker for immunotherapy response. Additionally, the presence of TILs, particularly cytotoxic T cells, has been linked to favorable outcomes in various cancers [172]. By identifying these biomarkers, clinicians can better stratify patients for immunotherapy and optimize treatment strategies based on individual tumor characteristics and immune profiles. Ongoing research aims to identify novel biomarkers and refine existing ones to further enhance the precision and efficacy of immunotherapy. Overall, the relationship between immune cells and specific treatment modalities for cancer underscores the importance of understanding and harnessing the immune system to develop effective therapeutic strategies against cancer.

## 7. Discussion

**Figure 6:**
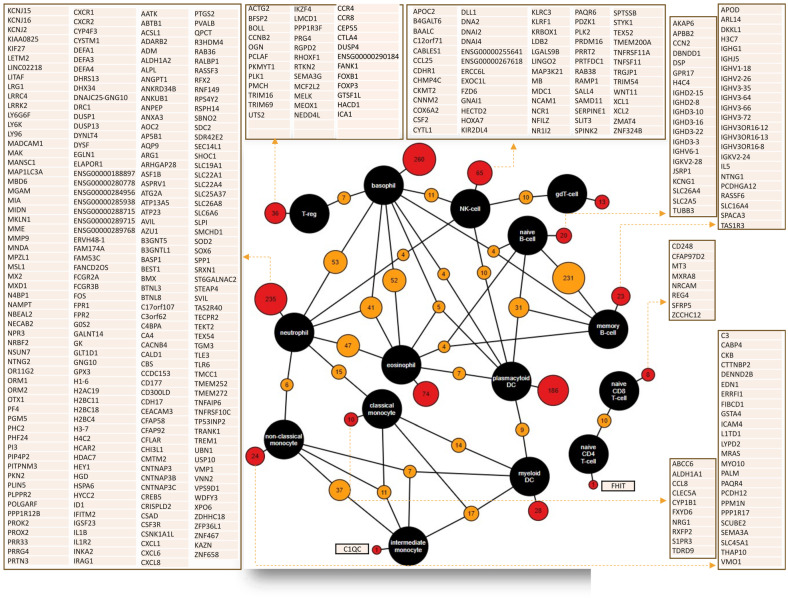
Genes involved in the regulation of important immune cells (i.e., immune-regulated genes, IRGs) that infiltrate tumor cells for exerting anti-tumor, pro-tumor, and other activities related to all aspects of tumor origin, progression, EMT, metastasis, and stemness.

The TME is a critical factor in the progression of metastasis and subsequent development of drug resistance leading to unfavorable prognosis and predictive outcomes. The primary constituents of TME are tumor cells and immune cells that have infiltrated the tumor, along with the stromal component. Several clinical interventions have demonstrated a correlation between ICI and the receptiveness to immunotherapy and the prognosis of cancer. The immune response inside the TME is widely acknowledged as a significant determinant of tumor aggressiveness, development, and the efficacy of immunomodulatory treatments. The characteristics of immune and inflammatory cells that infiltrate tumor cells serve as indicators of the host’s immune responses to the tumor [99,112,178,179]. We conducted a review investigation on the characteristics of immune and inflammatory cells that infiltrate tumors. Due to TME heterogeneity, immunosuppression, and off-target effects in cancer patients, many patients still have immunotherapy limitations [77,78]. Extensive research has been conducted on the prognostic indicators encompassing the number and composition of immune cells infiltrating tumors (Figure 6), along with the expression of cytokines and immune-related genes (IRGs). The group at high risk exhibited immune scores that were notably lower in comparison to the low-risk group, while patients with high immune scores tended to demonstrate more favorable prognosis. The augmented accumulation of CD8 T cells, CD4 T-helper cells, regulatory T cells, and proinflammatory macrophages of the M1 subtype, along with the diminished accumulation of immunosuppressive macrophages of the M2 subtype, are indicative of a more favorable prognosis for colorectal cancer (CRC) [180,181]. The presence of CD4+ T and CD8+ T cells within the tumor exhibited a positive association with the presence of CD8+ T cells, while demonstrating a negative association with the presence of regulatory T cells (%Treg) within the tumor. The group of patients at high risk exhibited a notable prevalence of M2 macrophages and a reduced prevalence of activated memory CD4 T cells, CD8 T cells, regulatory T cells, and M1 macrophages. These observations imply that the IRGs incorporated in combination may impact the prognosis by influencing the interaction with infiltrating immune cells (Figure 6).

A lack of a substantial link was seen between tumor-infiltrating myeloid cells and T cells, except for a strong correlation between tumor-infiltrating myeloid cells and the percentage of regulatory T cells. The results of this study indicate that the presence of myeloid cells and T cells within tumors is controlled by separate mechanisms as previously thought, and that the infiltration of certain subsets of myeloid cells or regulatory T cells is intimately interconnected [182]. However, with new insights it has now become obvious that various types of cells associated with tumors, including macrophages, dendritic cells, fibroblasts, and myeloid-derived suppressor cells, contribute to the recruitment of Tregs by the release of cytokines and chemokines [182]. As demonstrated by Liu et al. (2011), the production of CCL20 by TAMs has a role in the recruitment of CCR6+ Tregs in cases of colorectal cancer [183]. It is important to acknowledge that the infiltration of Tregs can potentially result in a favorable prognosis through the surveillance of inflammation linked to the neoplastic progression observed in some types of malignancies, such as colorectal and gastric cancers [54,183,184]. The tremendous impact of CCL1 on the accumulation of Tregs in the breast tumor environment is evident, as CCL1 serves as the functional ligand for CCR8 [185]. The expression of CCL1 is consistently increased in breast cancer, and there exists a positive association between CCL1 levels and the infiltration of CCR8+ regulatory T cells [182]. CCL1 is released by some myeloid cells within the TME to recruit Tregs expressing the CCR8 receptor. CCR8+ Tregs are characterized by the presence of the surface marker CD39, which plays a crucial role in the conversion of ATP to adenosine [182,186]. In addition, they secrete the anti-inflammatory cytokine IL10 and the apoptosis inducer granzyme-B [182]. These characteristics facilitate the ability of regulatory T cells (Tregs) to suppress the functions of effector T cells. The study conducted by Kuehnemuth and co-authors (2018) found a strong correlation between a high presence of CCR8+FOXP3+ Tregs and a worse prognosis in breast cancer patients [185]. Additionally, the study suggests that CCL1 could potentially serve as a viable therapeutic target for the treatment of breast cancer. Sporadic instances of renal cell carcinomas in humans exhibit heightened expression levels of CXCL9, CXCL10, and CXCL11, hence facilitating the recruitment of T cells that possess CXCR3 and CCR5 receptors. In contrast, it has been observed that human breast, ovarian, colorectal, and hepatocellular carcinomas exhibit the recruitment of CXCR3+FOXP3+ Tregs inside the TME [187].

Neutrophils comprise approximately 50–70% of the myeloid-derived leukocytes present in the circulating blood of humans. Their primary function is to participate in the innate immune response of the human body, specifically targeting and combating invading pathogens [188]. After being stimulated by cytokines, neutrophils gain the ability to polarize into either an anti-tumor (N1) or pro-tumor (N2) phenotype [189,190,191]. The immune profile of N1 TANs is distinguished by elevated levels of TNFα, CCL3, ICAM-1, and reduced levels of the Arginase axis. On the other hand, N2 neutrophils exhibit increased expression of chemokines such as CCL2, CCL3, CCL4, CCL8, CCL12, CCL17, CXCL1, CXCL2, IL-8/CXCL8, and CXCL16 [190].

Most inflammatory cells found in solid tumors are neutrophils, and there is a positive correlation between their high density within the tumor and the occurrence of metastasis at lymph node sites, as well as the tumor grade and stage axis [192]. The recruitment of neutrophils is influenced by both tumors and the tumor microenvironment, and it has been determined that TANs can modulate tumor progression or growth control [193]. Similar to macrophages, TANs can have either an anti-tumor effect (referred to as N1 neutrophils) or a pro-tumor effect (referred to as N2 neutrophils) [191,194]. The efficacy of N1 against tumor growth and metastasis is achieved through direct cytotoxicity or antibody-dependent cytotoxicity, as well as the activation of several innate and adaptive immune cells, such as T and B lymphocytes, NK cells, and DCs [195]. Moreover, N1 TANs demonstrate heightened NADPH oxidase activity, resulting in the generation of reactive oxygen species (ROS) that possess lethal effects on tumor cells [196]. In contrast, N2 TANs play a role in facilitating tumor growth and the spread of tumor cells by the secretion of enzymes that alter the ECM and proteins that stimulate the formation of new blood vessels (angiogenesis) and metastases [76,197,198]. Despite the significant recent advancements in characterizing the phenotype and roles of TANs in human cancer, there remains ongoing debate on the dual role of TANs in either limiting or encouraging the growth and metastatic spread of cancer cells. This controversy has given rise to several unresolved issues.

The findings from both univariate and multivariate survival studies demonstrated that the presence of tumor-infiltrating CD4+ T cells, CD8+ T cells, and the percentage of M1 macrophages were individually associated with the prognosis of OS and DFS [199,200,201]. Additionally, the presence of tumor-infiltrating pan-macrophages, M2 macrophages, neutrophils (Neus), and the percentage of regulatory T cells (%Tregs) were identified as independent prognostic factors for unfavorable OS and DFS outcomes. When the six variables (%M1, M2, Neus, CD4+ T, CD8+ T, and %Tregs) were combined and analyzed using survival analysis, it was observed that only one particular patient group (CD4+ T high and CD8+ T high, CD4+ T high and %Tregs low, CD8+ T high and %Tregs low, and % M1 high and M2 low) exhibited longer survival [200]. In contrast, the other three groups demonstrated shorter survival with comparable magnitudes. The findings of this study indicate that a comprehensive examination of various immunological and inflammatory cells that infiltrate tumors is crucial for obtaining a more accurate understanding of the impact of specific combinations of cells within the immune microenvironment. The aforementioned combinations were subsequently linked, resulting in the identification of two novel variables: tumor-infiltrating CD4+ T high/CD8+ T high/%Tregs low and %M1 high/M2 low [200]. The patient distribution was adjusted to address the imbalance, and the impact of immune-related components was accurately depicted. Indeed, the multivariate survival studies demonstrated that these two variables were autonomous predictors of OS and DFS, exhibiting hazard ratio values that surpassed those of individual sets of tumor-infiltrating cells. Hence, the variables of tumor-infiltrating CD4+ T high/CD8+ T high/%Tregs low and tumor-infiltrating %M1 high/M2 low are appropriate for assessing the immune microenvironment of PDAC [200].

Accumulating data have demonstrated that oxidative stress plays a pivotal role in modulating immune responses inside the TME, hence influencing the effectiveness of immunotherapeutic interventions. Reactive oxygen species play significant functions in augmenting antigen presentation, modulating immunological responses, and inhibiting immune-escape. This review provides a comprehensive overview of the prevailing cancer immunotherapeutic approaches and elucidates the intricate interplay between oxidative stress and the immunological TME. In the present review, our focus is directed at elucidating the fundamental processes responsible for the effectiveness of cancer immunotherapy, particularly in relation to its impact on oxidative stress, within the context of different malignancies. In addition, we emphasize the therapeutic implications of modulating oxidative stress as a means to enhance the efficacy of immunotherapies, hence potentially yielding advantageous outcomes in the realm of clinical application.

Thus, immunological and inflammatory cell infiltration into the tumor should serve as important markers for assessing the tumor immune milieu and monitoring the immune response in tumor tissues of both untreated and treated patients. The clinicopathological effects of distinct tumor-infiltrating cells and immune/inflammatory cell subsets must be assessed, as well as their interactions. Understanding the TME is crucial because some cell combinations might have major clinicopathological effects. Thus, studying distinct immunological and inflammatory cells that infiltrate tumors as indications of innate and adaptive immune responses in the same malignant tissue has great promise.

## 8. Future Perspectives: Implications in Therapeutics and Unanswered Questions

Collectively, the findings of this study indicate that the distribution patterns of various immune cell markers, specifically CD66b+ TANs, FoxP3+ Tregs, CD163+ TAMs, CD3, CD4 Th, CD8 CTLs, CD20, CD57, CD68, and CD163, have the potential to serve as prognostic indicators for individuals diagnosed with different cancers. The quantification of immune cell infiltration across different forms of cancer has revealed significant variation in the predictive and prognostic significance of these infiltrating immune cells. To date, conventional techniques for quantifying TIICs encompass flow cytometry, immunofluorescence, and others. For assessment and quantification purposes, some advanced technological platforms such as flow cytometry with radiomic classification, and computational models that employ tumor-infiltrating immune cells algorithms (referred to as ICCS model) to analyze high-throughput RNA-seq and other transcriptomics data are also available. This study discusses state-of-the-art computational methods for quantifying immune cells from cell mixture expression data using marker genes, GSEA, or deconvolution algorithms and immune cell expression signatures. Additional models such as Spatially Variant Immune Infiltration Scoring using SpatialVizScore and Nonnegative Tensor Factorization using ProTICS have also been discussed. These models demonstrate a high level of predictive and prognostic accuracy, contributing to a greater comprehension of the TME.

Most malignancies are caused by cancer stem cells (CSCs) or tumor-initiating cells (TICs). Although, TICs have a proven role in cancer diagnosis and prognosis; however, cancer therapies targeting TICs have been documented scarcely. Although, several immune cell-based therapies such as T-cell transfer therapy, adoptive cytokine-induced killer (CIK) cell therapy, chimeric antigen receptor (CAR) T-cell therapy, natural killer T (NKT) cell therapy, and others have been developed to tackle different types of human malignancies. To this end, TICs must be targeted to treat cancer since they are self-renewing, are tumorigenic, and are resistant to traditional therapy. TIC cell extraction and identification remain problematic and ambiguous despite advances in biology. This review does not clarify how TIICs link to CSCs and TICs, their metabolic properties, and new signaling pathways. We think a comprehensive debate on this may revolutionize cancer precision and individualized medicines.

## 9. Conclusions

The present review thoroughly investigates the potential of various combinations of tumor-infiltrating immunological and inflammatory cells as indicators of the immune microenvironment across different cancer types. It offers a comprehensive perspective on TIICs and provides novel insights into the molecular mechanisms for enhancing existing diagnostic, prognostic, and therapeutic approaches. TIICs hold promise for bridging conventional therapies with modern immunotherapies. However, the relationship between IRGs, TIICs, and their response to neoadjuvant chemotherapy (NAC) in diverse malignancies remains underexplored. The interplay among TIICs, IRGs, and NAC underscores the importance of combining NAC with immunotherapy to enhance clinical outcomes. Additionally, the successful application of single-cell sequencing techniques in determining TIIC composition has yet to be demonstrated. Furthermore, our review systematically presents the infiltration of immune cells into the tumor core across different cancers and examines the resulting functional implications. We scrutinize the associations between T cells, NK cells, B cells, macrophages, and clinical characteristics, while also delineating the distinct activities of T cells and macrophages upon infiltration into various tumor areas. By compiling relevant information on TIICs, the TME, TMB, and other parameters, we discuss various prediction models based on immune cells that accurately forecast survival outcomes and the effects of chemotherapy and other treatments. Implementing this approach in clinical practice could aid in identifying patients unsuitable for CT treatments and identifying suitable candidates for immunotherapy. Moreover, several techniques have demonstrated the potential to genetically alter TIICs to enhance their cytotoxicity, tumor homing, or reduce T cell exhaustion. However, as of now, no therapy based on TIICs has been approved by the FDA, although numerous clinical trials are underway at various stages. Therefore, investigating the fraction of TIICs holds promise for advancing research on the tumor microenvironment and evaluating adjuvant chemotherapy, NAC, and other targeted cancer treatments.

## Figures and Tables

**Table 1:** A list of potential immune biomarkers and their applications in the prognosis of different cancers.

S. No.	Immune Biomarker (Official Gene Symbol)	High Expression— Unfavorable Prognosis	High Expression— Favorable Prognosis	Reference
1	*PSMB8*	LAML, LAUD, PDAC	BLCA, BRCA, MESO	[133]
2	*LCN2*	BLCA, KIRC, GBM	BRCA, ESCA, LGG, THCA	[134]
3	*TIGIT*	KIRC, KIRP, LGG, UVM	BRCA, CECS, HNSC, SKCM	[135]
4	*HMGBs*	BLCA, PRAD	GC	[136] [143] [137] [144]
5	*STC2*	BLCA, COAD, ESCA HNSC, KIRP, LIHC LUAD, MESO, SARC THYM	HNSCC, BRCA, LGG	[145]
6	*FN1*	BRCA		[146]
7	*PD-1 and PD-L1*	RCC, BRCA	HNSCC	[147] [148]
8	*KIF2C*	GBM, CRC, GC		[149] [150] [151]
9	*NUSAP1*	TNBC		[152]
10	*TLR3*		ESCC	[153]
11	*INHBA*	CESC		[154]
12	*EPHA3*		BLCA	[155]
13	*CD73*	GBOV		[156]
14	*HSPB11*	HCC		[138]
15	*JAM2*		BRCA	[141]
16	*OX40L*		HNSCC	[148]
17	*PDGFRB*		HNSCC	[148]
18	*TMCO3*	LIHC		[157]
19	*IGF2BP2*	AML		[158]
20	*ACTA2*	BLCA		[159]
21	*TPM1*	BLCA		[159]
22	*ACTC1*	BLCA, GBM	PDAC	[159] [160] [161]
23	*ACTN1*	BLCA		[159]
24	*PPARG*		BLCA	[159]
25	*COL3A1*	BLCA		[159] [162]
26	*IGFBP3*	CRC		[163]
27	*EPCAM*	BLCA	LAUD BRCA BLCA ESCC	[164] [165]
28	*TPM1*		GC	[142]
29	*SDC1*	BRCA	ICC, CRC	[166] [167] [139]
30	*NGAL*	BRCA		[168]
31	*DZIP1*	GC		[140]
32	*SLC25A25-AS1*	PRAD		[169]

AML, acute myeloid leukemia; BLCA, bladder cancer; BRCA, breast cancer; CESC, cervical cancer; CHOL, cholangiocarcinoma; COAD, colon cancer; ESCA, esophageal cancer; GBM, glioblastoma; gingivobuccal oral cancer, GBOC; HNSC, head and neck cancer; KICH, kidney chromophobe; KIRC, kidney clear cell carcinoma; KIRP, kidney papillary cell carcinoma; LGG, lower grade glioma; LIHC, liver cancer; LUAD, lung adenocarcinoma; LUSC, lung squamous cell carcinoma; MESO, mesothelioma; OV, ovarian cancer; ovarian serous cyst adenocarcinoma (OSCA); PDAC, pancreatic cancer; PRAD, prostate cancer; READ, rectal cancer; SARC, sarcoma; SKCM, melanoma; THCA, thyroid cancer; THYM, thymoma; TNBC, triple-negative breast cancer; UCEC, endometrioid cancer; UCS, uterine carcinosarcoma; UVM, uveal/ocular melanoma.

## Abbreviations

AML, acute myeloid leukemia; BLCA, bladder cancer; BRCA, breast cancer; CESC, cervical cancer; CHOL, cholangiocarcinoma; COAD, colon cancer; ESCA, esophageal cancer; GBM, glioblastoma; gingivobuccal oral cancer, GBOC; HNSC, head and neck cancer; KICH, kidney chromophobe; KIRC, kidney clear cell carcinoma; KIRP, kidney papillary cell carcinoma; LGG, lower grade glioma; LIHC, Liver Cancer; LUAD, Lung Adenocarcinoma; LUSC, Lung Squamous Cell Carcinoma; MESO, mesothelioma; OV, ovarian cancer; ovarian serous cyst adenocarcinoma (OSCA); PDAC, pancreatic cancer; PRAD, prostate cancer; READ, rectal cancer; SARC, sarcoma; SKCM, melanoma; THCA, thyroid cancer; THYM, thymoma; TNBC, triple-negative breast cancer; UCEC, endometrioid cancer; UCS, uterine carcinosarcoma; UVM, uveal/ocular melanoma
